# Supplementary material for: The Incidence and Impact of In-Hospital Bleeding in Patients with Acute Coronary Syndrome during the COVID-19 Pandemic
Source: J Clin Med. 2022 May 22;11(10):2926. doi: 10.3390/jcm11102926 (PMC9146584; doi:10.3390/jcm11102926)
Supplement: Supplementary file 1 [file jcm-11-02926-s001.zip › new tables.pdf]

Supplementary Table S1: Baseline characteristics of patients admitted for acute coronary syndrome before and during COVID-19 pandemic.

	Before COVID-19 Pandemic (n = 2142)	During COVID-19 Pandemic (n = 709)	P
Age	67±12,47	67±11,862	0,805
BMI (kg/m^2)	26±4	26±4	0,74
Female Sex	26,10%	26,00%	0,933
Hypertension	69,20%	69,60%	0,833
Diabetes Mellitus	27,70%	28,80%	0,57
Dyslipidemia	50,20%	50,10%	0,939
Smoking	27,90%	30,60%	0,165
Ex-Smoking	18,20%	14,00%	**0,012**
Atrial Fibrillation (All Forms)	9,60%	8,80%	0,514
History Of Heart Failure	5,20%	4,90%	0,794
Valve Disease (more than mild)	0,40%	0%	**0,012**
COPD	8,10%	8,20%	0,899
Respiratory/Pulmonary Disease	0,50%	0,40%	0,765
Neurological Disease	0,60%	0,60%	0,99
Chronic Kidney Disease (GFR < 60 ml/min)	11,50%	13,20%	0,225
Hemorrhagic Diatesis	1,10%	0%	**0,005**
Thrombosis Diatesis (PVT)	1,20%	0,40%	0,082
Anemia	0,50%	0,30%	0,51
Inflammatory/Infective Disease	1,80%	1,10%	0,237
Previous Oncological Disease	1,40%	0,60%	0,075
Previous PCI	22,60%	21,80%	0,672
Previous CABG	5,60%	4,80%	0,429
Previous MI	20,40%	19,10%	0,463
Previous Stroke/TIA	4,30%	4,50%	0,833
Atypical Symptoms	13,40%	14,90%	0,312
Dyspnea	13,10%	13,40%	0,842
Fever	1,70%	5,10%	**<0,001**
Heart Failure (at the presentation)	13,00%	12,50%	0,717
Killip Class > 1	28,60%	37,50%	**<0,001**
EF (%; at presentation)	48,6±9,8	47,1±9,9	**0,001**
Cardiac Arrest before Cathlab	3,50%	3,00%	0,456
STEMI	43,30%	47,20%	0,065
NSTEMI	39,30%	38,10%	0,576
Unstable Angina	12,90%	9,60%	**0,02**
Total Thrombotic occlusion	37,40%	37,70%	0,919
Thrombus Aspiration	17,30%	14,50%	0,08
Number of stent implanted	0,82±0,39	0,80±0,45	0.49
Fibrinolysis	0,30%	0%	0,167

GP IIB/IIIA use	11,00%	9,40%	0,216
Any Ventricular Support	0,1%	0,6%	0.004

BMI= body mass index; COPD=chronic obstructive pulmonary disease ;PCI= percutaneous coronary intervention ;CABG= coronary artery bypass graft; MI=myocardial infarction; TIA=transient ischemic attack; EF=ejection fraction; STEMI=ST-segment elevated myocardial infarction; NSTEMI= non ST-segment elevated myocardial infarction; GP= glycoprotein.

Supplementary Table S2: Baseline characteristics of patients admitted for acute coronary syndrome during the COVID-19 pandemic first wave with positive and negative COVID-19 swab.

	Negative COVID-19 swab (n = 639)	Negative COVID-19 swab (n = 70)	P
Age	67±12	68±12	0,34
BMI (kg/m2)	27±4	24±4	<0,001
Female Sex	26,30%	22,90%	0,534
Hypertension	70,10%	65,70%	0,451
Diabetes Mellitus	27,40%	41,40%	0,014
Dyslipidemia	51,50%	37,10%	0,023
Smoking	32,10%	17,10%	0,01
Ex-Smoking	14,10%	13,00%	0,807
Atrial Fibrillation (all forms)	8,60%	10,10%	0,669
History of Heart Failure	4,60%	7,20%	0,341
COPD	8,30%	7,10%	0,728
Respiratory/Pulmonary Disease	0,50%	0,00%	0,566
Neurological Disease	0,50%	1,40%	0,309
Chronic Kidney Disease	12,60%	18,60%	0,161
Thrombosis Diatesis (PVT)	0,50%	0,00%	0,566
Anemia	0,20%	1,40%	0,057
Inflammatory/Infective Disease	1,30%	0,00%	0,346
Previous Oncological Disease	0,50%	1,40%	0,309
Previous PCI	22,40%	17,10%	0,316
Previous CABG	4,10%	11,40%	0,007
Previous MI	20,00%	11,40%	0,084
Previous Stroke/TIA	4,30%	7,10%	0,27
Atypical symptoms	14,80%	15,90%	0,798
Dyspnea	12,10%	24,60%	0,004
Fever	3,20%	22,10%	<0,001
Heart Failure (at the presentation)	11,80%	18,80%	0,092
Killip Class > 1	36,60%	45,70%	0,136
EF (%; at presentation)	48±10	44±6	0,006
Cardiac arrest before Cathlab	2,80%	4,30%	0,491
STEMI	44,90%	68,60%	<0,001
NSTEMI	40,40%	17,10%	<0,001
Unstable Angina	10,20%	4,30%	0,112
MINOCA	3,30%	4,30%	0,661

TakoTsubo Syndrome	1,70%	7,10%	0,004
Total Thrombotic occlusion	35,40%	58,00%	<0,001
Thrombus Aspiration	14,80%	11,60%	0,472
Number of stent implanted	0,8±0,45	0,84±0,47	0,488
GP IIB/IIIA use	9,6%	7,2%	0,523
Any Ventricular Support	0,50%	1,40%	0,309
Arrhythmic complications during procedure	3,80%	11,60%	0,003
Intra-hospital Arrhythmic Complications	3,20%	1,40%	0,424
Intra-hospital Mechanical Complications	0,80%	2,90%	0,094
Intra-hospital TIMI Major bleeding	0,90%	0,00%	0,421
Intra-hospital TIMI Minor bleeding	2,50%	4,40%	0,36
Total Bleeding	3,40%	4,30%	0,717
Deaths during hospitalization	1,60%	7,10%	0,002

BMI= body mass index; COPD=chronic obstructive pulmonary disease ;PCI= percutaneous coronary intervention ;CABG= coronary artery bypass graft; MI=myocardial infarction; TIA=transient ischemic attack; EF=ejection fraction; STEMI=ST-segment elevated myocardial infarction; NSTEMI= non ST-segment elevated myocardial infarction; GP= glycoprotein.

Supplementary Table S3: Baseline characteristics of the population with or without in-hospital bleeding based on COVID-19 swab result.

	Negative COVID-19 swab **(n = 639)**			Positive COVID-19 swab **(n = 70)**		
	No Bleed	Bleed	P	No Bleed	Bleed	P
Age	66.8±12.3	69±12.9	0,21	68.1±12.3	70.3±4	0,24
BMI (kg/m2)	26 ± 4	27 ± 4	0.77	24 ± 4	24 ± 2	0.93
Female Sex	25,70%	42,90%	0.08	23,90%	0,00%	0.33
Hypertension	69,70%	81,00%	0.27	64,20%	100,00%	0.20
Diabetes mellitus	27,40%	28,60%	0.90	41,80%	33,30%	0.77
Dyslipidemia	52,10%	33,30%	0.09	35,80%	66,70%	0.30
Smoking	32,20%	28,60%	0.72	17,90%	0,00%	0.42
Ex-smoking	14,30%	9,50%	0.54	12,10%	33,30%	0.28
Atrial Fibrillation	8,40%	14,30%	0.34	9,10%	33,30%	0.17
History of Heart Failure	4,50%	9,50%	0.28	6,10%	33,30%	0.07
Vascular diseases	1,30%	9,50%	0.003	0,00%	0,00%	NA
COPD	8,30%	9,50%	0.84	7,50%	0,00%	0.62
Respiratory/Pulmonary disease	0,50%	0,00%	0.75	0,00%	0,00%	
Chronic kidney disease	12,20%	23,80%	0.11	17,90%	33,30%	0.50
Thrombotic diathesis	0,50%	0,00%	0.75	0,00%	0,00%	
Anemia	0,20%	0,00%	0.85	1,50%	0,00%	0.83
Inflammatory/Infective disease	1,30%	0,00%	0.60	0,00%	0,00%	
Previous oncological disease	0,30%	4,80%	0.003	1,50%	0,00%	0.83

Previous PCI	22,60%	14,30%	0.36	16,40%	33,30%	0.45
Previous CABG	3,90%	9,50%	0.20	10,40%	33,30%	0.22
Previous MI	20,20%	14,30%	0.50	10,40%	33,30%	0.22
Previous Stroke/TIA	4,40%	0,00%	0.32	7,50%	0,00%	0.62
Typical symptoms	74,10%	81,00%	0.48	78,80%	66,70%	0.62
Atypical symptoms	14,80%	14,30%	0.95	15,20%	33,30%	0.40
Intermittent Angina	13,70%	9,50%	0.58	13,60%	0,00%	0.49
Dyspnea	12,20%	9,50%	0.71	22,70%	66,70%	0.08
Respiratory impairment	6,00%	0,00%	0.25	15,20%	66,70%	0.02
Fever	3,20%	4,80%	0.68	21,50%	33,30%	0.63
Heart Failure (at the presentation)	11,70%	14,30%	0.71	19,70%	0,00%	0.07
Killip Class (at presentation) > 1	98,10%	100,00%	0.16	100,00%	100,00%	0.74
Night Presentation	20,50%	14,30%	0.49	19,40%	0,00%	0.40
EF (%; at presentation)	48 ± 10	41 ± 9	0.005	44 ± 6	52 ± 5	0.09
Time Door to Balloon (minutes)	257 ± 609	150 ± 351	0.48	34 ± 49	35 ± 7	0.98
Time Symptoms to Cath-lab door (minutes)	1360 ± 3808	651 ± 967	0.44	579 ± 2622	827 ± 1363	0.87
Time Symptoms to Emergency call (minutes)	875 ± 2806	925 ± 1944	0.94	428 ± 1438	30	0.78
Cardiac arrest before cathlab	2,60%	9,50%	0.06	4,50%	0,00%	0.71
STEMI	44,30%	61,90%	0.11	70,10%	33,30%	0.18
NSTEMI	40,80%	28,60%	0.26	14,90%	66,70%	0.02
Unstable Angina	10,20%	9,50%	0.92	4,50%	0,00%	0.71
Thrombotic occlusion	34,90%	50,00%	0.16	59,10%	33,30%	0.37
Number of stents implanted	0,80 + 0,45	0,86 + 0,48	0.56	0.85 + 0.45	0.67 + 0.57	0.79
GP IIB/IIIA use	9,90%	0,00%	0.13	7,60%	0,00%	0.62
Thrombus Aspiration	15,00%	9,50%	0.49	12,10%	0,00%	0.52
Any Ventricular Support	2,80%	9,50%	0.21	7,50%	0,00%	0.89
Mechanical Complications (Intra-Procedural)	2,50%	14,30%	0.001	1,50%	0,00%	0.83

BMI= body mass index; COPD=chronic obstructive pulmonary disease ;PCI= percutaneous coronary intervention ;CABG= coronary artery bypass graft; MI=myocardial infarction; TIA=transient ischemic attack; EF=ejection fraction; STEMI=ST-segment elevated myocardial infarction; NSTEMI= non ST-segment elevated myocardial infarction; GP= glycoprotein.

Supplementary Table S4: Univariate analysis for TIMI major or minor bleeding.

	Hazard ratio (95% CI)			P value
		Lower	Upper	
Age	1.013	0.995	1.03	0.159

Female gender	1.892	1.226	2.921	0.004
Hypertension	1.15	0.718	1.843	0.561
Diabetes mellitus	0.956	0.596	1.534	0.853
Dyslipidemia	0.617	0.398	0.956	0.031
Previous PCI	1.122	0.68	1.851	0.652
Previous CABG	1.09	0.442	2.689	0.852
Previous MI	1.286	0.779	2.121	0.325
Smoking	0.823	0.503	1.346	0.438
Atrial Fibrillation	1.549	0.856	2.804	0.148
Heart Failure at the presentation	1.24	0.714	2.154	0.445
History of Heart Failure	1.618	0.746	3.507	0.223
Previous Stroke/TIA	1.839	0.849	3.988	0.123
COPD	1.762	0.956	3.249	0.069
Chronic kidney disease	2.133	1.292	3.523	0.003
Anemia	6.595	2.075	20.963	0.001
Peripheral vascular diseases	2.786	1.018	7.629	0.046
Valve disease	6.073	1.482	24.882	0.012
Respiratory/Pulmonary disease	1.895	0.263	13.673	0.526
Inflammatory/Infective disease	0.653	0.091	4.691	0.672
Thrombotic diathesis	1.751	0.429	7.154	0.435
Hemorrhagic diathesis	3.602	1.137	11.416	0.029
Previous oncological disease	3.991	1.607	9.913	0.003
Neurological disease	2.926	0.715	11.981	0.135
STEMI presentation	1.003	0.656	1.534	0.989
NSTEMI presentation	1.12	0.728	1.723	0.605
Unstable Angina presentation	0.575	0.232	1.424	0.232
MINOCA presentation	1.12	0.354	3.545	0.847
Cardiac arrest before Cathlab	1.847	0.802	4.253	0.149
Fever at presentation	1.553	0.566	4.259	0.393
Killip Class at presentation	1.398	1.119	1.747	0.003
EF (%) at presentation)	0.972	0.952	0.993	0.011
Echo Wall motion abnormalities	2.46	1.131	5.35	0.023
Number of diseased vessels	0.97	0.862	1.091	0.607
Fibrinolysis	4.585	0.635	33.115	0.131
GP IIB/IIIA use	0.871	0.436	1.738	0.694
Mechanical Complications in cathlab	5.692	2.729	11.872	< 0.001
Inotropes at presentation and/or during procedure	1.88	0.988	3.579	0.054
Cardiac Arrest during procedure	1.226	0.301	4.993	0.776

BMI= body mass index; COPD=chronic obstructive pulmonary disease ;PCI= percutaneous coronary intervention ;CABG= coronary artery bypass graft; MI=myocardial infarction; TIA=transient ischemic attack; EF=ejection fraction; STEMI=ST-segment elevated myocardial infarction; NSTEMI= non ST-segment elevated myocardial infarction; GP= glycoprotein.

Supplementary Table S5: Multivariate analysis for TIMI major or minor bleeding.

	**Hazard ratio (95% CI)**			**P value**
Female gender	2.053	1.291	3.266	0.002

Dyslipidemia	0.541	0.337	0.87	0.011
Chronic kidney disease	1.85	1.089	3.141	0.023
Anemia	5.538	1.519	20.191	0.01
Previous oncological disease	2.655	0.966	7.296	0.058
Killip Class (at presentation)	1.309	1.038	1.651	0.023
Echo Wall motion abnormalities	2.092	0.895	4.89	0.088
Mechanical Complications in cathlab	4.844	2.179	10.77	< 0.001
