# Supplementary figures and images for: The Incidence and Impact of In-Hospital Bleeding in Patients with Acute Coronary Syndrome during the COVID-19 Pandemic
Source: J Clin Med. 2022 May 22;11(10):2926. doi: 10.3390/jcm11102926 (PMC9146584; doi:10.3390/jcm11102926)

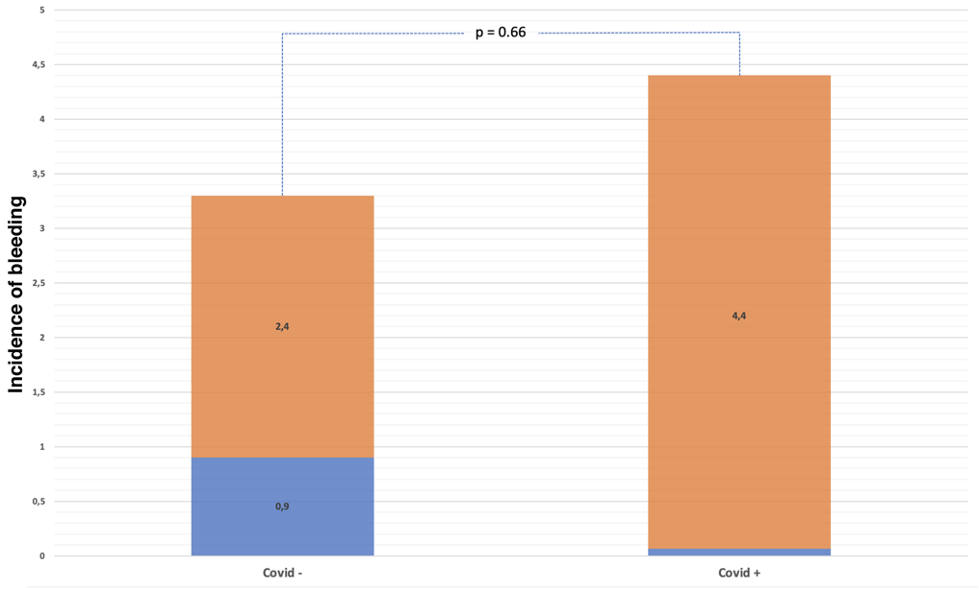

Supplement: Supplementary file 1 [file jcm-11-02926-s001.zip › Supplementary Figure S1.png]

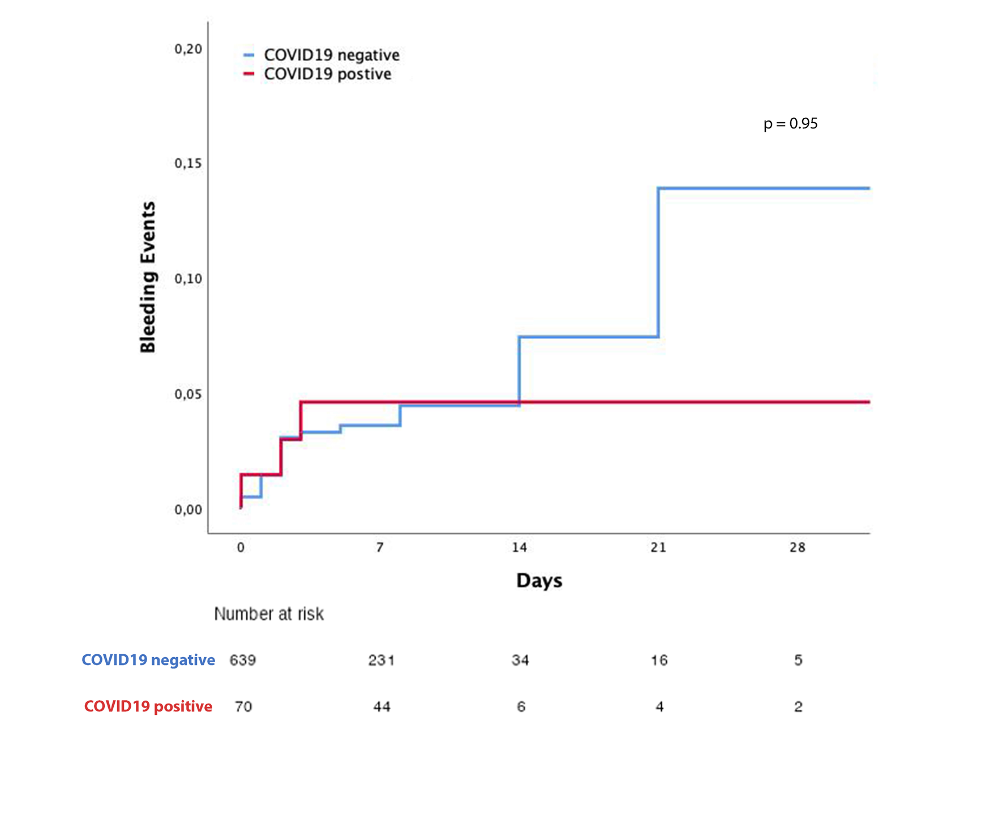

Supplement: Supplementary file 1 [file jcm-11-02926-s001.zip › Supplementary Figure S2.png]

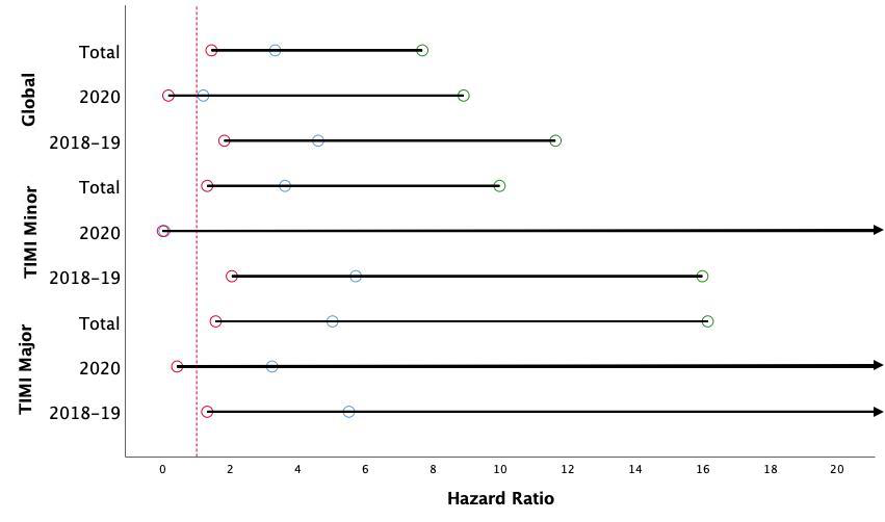

Supplement: Supplementary file 1 [file jcm-11-02926-s001.zip › Supplementary Figure S3.png]
